# Supplementary material for: GenEpi: gene-based epistasis discovery using machine learning
Source: BMC Bioinformatics. 2020 Feb 24;21:68. doi: 10.1186/s12859-020-3368-2 (PMC7041299; doi:10.1186/s12859-020-3368-2)
Supplement: Supplementary file 1 — Additional file 1. Supplementary information for literature survey of the genetic features selected by GenEpi and the formulas for linkage disequilibrium estimation. [file 12859_2020_3368_MOESM1_ESM.docx]

GenEpi: Gene-based Epistasis Discovery Using Machine Learning.

**Additional file 1**

## The formulas for linkage disequilibrium estimation

Linkage disequilibrium (LD) was first used in 1960 by (Lewontin and Kojima, 1960) and refers to the non-random association of alleles at different loci in a given population. Loci are said to be in linkage disequilibrium when the frequency of association of their different alleles is higher or lower than what would be expected if the loci were independent and associated randomly. Linkage disequilibrium or equilibrium depends on the occurrence of recombination, which is facilitated by chromosomal crossover during meiosis and that would result in descendants having different combinations of genes and occasionally produce new allele. We can consider recombination as a shuffling procedure, and in a younger population such as European, which has fewer shuffling times so their SNP features may have more dependency, resulting in larger LD blocks. In machine learning, we can take the advantage of LD to reduce the dimension of SNP features. In this regard, we followed the study of Lewontin (Lewontin, 1964) to estimate LD using Equation S1-4 to reduce the redundant SNP features. Equation S1 calculates the coefficient of linkage disequilibrium *D*, where *p_A_* is the proportion of chromosomes with the allele type A at one locus, *p_B_* is the proportion with the allele type B at another locus, and *p_AB_* is the proportion of chromosomes with both A and B on the same chromosome. In genotyped data, we do not have phased information to calculate *p_AB_*, therefore we used the Hill’s Expectation-Maximization (EM) algorithm approach (Hill, 1974) to predict the haplotype and estimate *p_AB._*_._

(Equation S1)

$$D = p_{AB}-p_{A}p_{B}$$

The formulas of normalized *D* are as Equation S2 and S3

(Equation S3)

(Equation S2)

$D' = D/{D_{\min}}$ , where

$D_{\min}=\left\{ \begin{aligned} \max\left\{ -\left( p_{A}p_{B} \right),-\left( 1-p_{A} \right)\left( 1-p_{B} \right) \right\} \mathrm{when} D<0 \\ \min\left\{ p_{A}\left( 1-p_{B} \right), \left( 1-p_{A} \right)p_{B} \right\} \mathrm{when} D>0 \end{aligned} \right.$

Equation S4 is the formula of correlation coefficient *r^2^*, which is an alternative to *D’,* referring to the correlation coefficient between pairs of loci.

(Equation S4)

$$r^{2} = \frac{D^{2}}{p_{A} \left( 1-p_{A} \right) p_{B} \left( 1-p_{B} \right)}$$

## Literature survey of the genetic features selected by GenEpi

**S.2.1 cross-gene epistasis**

GenEpi picked out only one cross-gene epistasis, which is *MICB * TOB2*. MIC proteins are induced on the cell surface by stress and are ligands of a common activatory natural killer-cell receptor (*NKG2D*) (Vivier, et al., 2002). They thus have roles in antimicrobial defense, tumor suppression and autoimmune-like diseases (Collins, 2004). Quiroga et al. has found that the human MHC class I chain-related genes (*MICA* and *MICB*) are located within the HLA region that has stratified association with AD in the cohort of the Oxford Project to Investigate Memory and Ageing (OPTIMA) even though the association did not survive the correction for multiple testing (Quiroga, et al., 2009). *TOB2* has been reported as a most up-regulated gene (Mirza and Rajeh, 2017) by expression analysis on multiple AD expression datasets from the GEO database to identify significant genes associated with electrophysiological pathways and attempted determination of interconnected canonical molecular pathways. The interaction of *MICB* and *TOB2* suggests a not-yet-explored biological process in AD pathogenesis. However, the significant associations of these genes in genome-wide association studies associated with immune system diseases and neurological diseases (see GWAS Catalog, 22561518, 29083406, 28540026), warrant future investigation.

**S.2.2 single-gene epistasis**

There are several possible modes of action accounting for intramolecular SNP-SNP interactions identified in this study. First: a synergistic regulation of transcription. For example, rs12095538 resides in the eQTL of STY6 and rs2774308 resides in a DNAse region of STY6. It is very possible that variants in both rs12095538 and rs2774308 synergistically affect the expression of STY6. SYT6 encodes protein control neurotransmitter release. It has been shown that the expression of SYT6 is significantly down regulated in AD (Saura, et al., 2015), supporting the causative role of the inherited rs12095538 and rs2774308 haplotype in AD progression. Likewise, rs56233035 and rs3678 reside in the LD blocks containing the eQTL and DNAse region of CACNA1E, respectively. CACNA1E encodes a subunit of CaV1.2 calcium channel. Its expression level in reactive astrocytes is associated with amyloid-β plaques formation in an AD mouse model (Daschil, et al., 2013). Another examples are the interactions between rs12926153 and rs12922908, and rs9652600 and rs12922908 that are within DNAse regions controlling the expression of CLEC16A in brain. CLEC16A encodes a lectin receptor involved in inflammation process and is differentially expressed in AD brains (Porcellini, et al., 2010). Lastly, rs2052573 and rs34580133 reside in DNAse regions within the LINC00299 and thus may synergistically control the expression of this long non-coding RNA that has been implicated in brain development (Talkowski, et al., 2012).

Second: a synergestic interaction between transcriptional and post-transcriptional regulation. For example, rs9344977, in an intron of BACH2 containing cis-regulatory elements, may affect the expression of BACH2, whereas rs56148686 in another intron of BACH2 without any cis-regulatory element. We suspect that rs56148686 may interact with s9344977 through a post-transcriptional mechanism such as RNA stability, RNA splicing, or microRNA binding. BACH2 encodes a transcription factor involving cellular responses toward oxidative stress. Interestingly, BACH2 is upregulated in cultured human neuroblastoma cells upon exposure to Alzheimer Amyloid β (Uhrig, et al., 2009). Similar mechanisms may apply to interaction pairs rs12189429-rs6881360 and rs12187423-rs6881360 that presumably regulating the abundance of ADAMTS12 mRNA. The importance of ADAMTS12 in AD is supported by another independent study using same ANDI dataset (Wang, et al., 2015). Third: an intramolecular SNP pairs modulating the expression of two separate neighboring genes. For example, rs11675339 and rs2710687 of VSNL1 appear to control the expression of GEN1 and SMC6 respectively. We also found that VSNL (also known as VILIP-1) has strong evidences to AD (Babic Leko, et al., 2016; Kirkwood, et al., 2016; Luo, et al., 2013). GEN1 and SMC6 play pivotal roles in repairing double-strand breaks of genomic DNA. Interestingly, rDNA instability, a result of failures to repair double-strand breaks of genomic DNA, has been implicated in AD (Pietrzak, et al., 2011). Most of the single-gene epistasis selected by GenEpi can be explained by these three possible modes and only two of the significance SNP-SNP interactions are not immediately clear at this moment. For example, rs12366151 resides in the eQTL of MICALCL, an AD associated gene (Rouillard, et al., 2016) but the interacting rs10831829 resides in the intron of PARVA, a gene not-yet-reported to be associated with AD. Similarly, rs2421701 and rs200512701 reside in two eQTLs regulating TNKS2 expression. However, the ADP-ribose polymerase functionally contributes to AD is not clear.

**Supplemental Tables**

**Table S1.**The summary of the number of SNPs, genes and features in each step of GenEpi .

| Step | 0 | 1 | 2 | 3 | 4 | 5 |
| --- | --- | --- | --- | --- | --- | --- |
| Function | Raw data | Download UCSC Database | Estimate LD Blocks | Split Features By Genes | Select Single- Gene Epistasis | Select Cross- Gene Epistasis |
| Number of SNP | 12,809,667 | 12,809,667 | 12,102,888 | 4,916,249 | 44,865 | 24 |
| Number of Gene | - | 22,376 | 22,376 | 20,206 | 7,933 | 12 |
| Number of Feature | - | - | - | - | 34,689 | 14 |

**References**

Babic Leko, M.*, et al.* Predictive Value of Cerebrospinal Fluid Visinin-Like Protein-1 Levels for Alzheimer's Disease Early Detection and Differential Diagnosis in Patients with Mild Cognitive Impairment. *J Alzheimers Dis* 2016;50(3):765-778.

Collins, R.W. Human MHC class I chain related (MIC) genes: their biological function and relevance to disease and transplantation. *Eur J Immunogenet* 2004;31(3):105-114.

Daschil, N.*, et al.* CaV1.2 calcium channel expression in reactive astrocytes is associated with the formation of amyloid-beta plaques in an Alzheimer's disease mouse model. *J Alzheimers Dis* 2013;37(2):439-451.

Hill, W.G. Estimation of linkage disequilibrium in randomly mating populations. *Heredity* 1974;33(2):229.

Kirkwood, C.M.*, et al.* Altered Levels of Visinin-Like Protein 1 Correspond to Regional Neuronal Loss in Alzheimer Disease and Frontotemporal Lobar Degeneration. *J Neuropathol Exp Neurol* 2016;75(2):175-182.

Lewontin, R. and Kojima, K.i. The evolutionary dynamics of complex polymorphisms. *Evolution* 1960;14(4):458-472.

Lewontin, R.C. The Interaction of Selection and Linkage. I. General Considerations; Heterotic Models. *Genetics* 1964;49(1):49-67.

Luo, X.*, et al.* CSF levels of the neuronal injury biomarker visinin-like protein-1 in Alzheimer's disease and dementia with Lewy bodies. *J Neurochem* 2013;127(5):681-690.

Mirza, Z. and Rajeh, N. Identification Of Electrophysiological Changes In Alzheimer's Disease: A Microarray Based Transcriptomics And Molecular Pathway Analysis Study. *CNS Neurol Disord Drug Targets* 2017.

Pietrzak, M.*, et al.* Epigenetic silencing of nucleolar rRNA genes in Alzheimer's disease. *PLoS One* 2011;6(7):e22585.

Porcellini, E.*, et al.* Alzheimer's disease gene signature says: beware of brain viral infections. *Immun Ageing* 2010;7:16.

Quiroga, I.*, et al.* Association study of MICA and MICB in Alzheimer's disease. *Tissue Antigens* 2009;74(3):241-243.

Rouillard, A.D.*, et al.* The harmonizome: a collection of processed datasets gathered to serve and mine knowledge about genes and proteins. *Database (Oxford)* 2016;2016.

Saura, C.A., Parra-Damas, A. and Enriquez-Barreto, L. Gene expression parallels synaptic excitability and plasticity changes in Alzheimer's disease. *Front Cell Neurosci* 2015;9:318.

Talkowski, M.E.*, et al.* Disruption of a large intergenic noncoding RNA in subjects with neurodevelopmental disabilities. *Am J Hum Genet* 2012;91(6):1128-1134.

Uhrig, M.*, et al.* New Alzheimer amyloid beta responsive genes identified in human neuroblastoma cells by hierarchical clustering. *PLoS One* 2009;4(8):e6779.

Vivier, E., Tomasello, E. and Paul, P. Lymphocyte activation via NKG2D: towards a new paradigm in immune recognition? *Curr Opin Immunol* 2002;14(3):306-311.

Wang, W.*, et al.* A Multi-Marker Genetic Association Test Based on the Rasch Model Applied to Alzheimer's Disease. *PLoS One* 2015;10(9):e0138223.
